# Two binding sites are better than one

**DOI:** 10.7554/eLife.110917

**Published:** 2026-04-01

**Authors:** Mackenzie K Scott, John E Burke

**Affiliations:** 1 Department of Biochemistry and Microbiology, University of Victoria Victoria Canada; 2 University of Victoria Genome BC Proteomics Centre Victoria Canada; 3 Department of Biochemistry and Molecular Biology, University of British Columbia Vancouver Canada

**Keywords:** membrane biology, VPS34, Rab1, Rab5, class III PI3K signalling pathway, eukaryotic evolution, Human, *S. cerevisiae*, *S. pombe*

## Abstract

The reasons why two multiprotein complexes – VPS34 complex I and VPS34 complex II – are activated by different Rab proteins are becoming clearer.

**Related research article** Špokaitė S, Ohashi Y, Bourguet M, Dessus AN, Williams RL. 2026. A novel RAB5 binding site in human VPS34-CII that is likely the primordial site in eukaryotic evolution. *eLife* **15**:RP110040. doi: 10.7554/eLife.110040.

Thousands of chemical reactions take place in cells every second, and many of them must take place at the correct location on a specific intracellular membrane. An enzyme called VPS34, also known as class III PI3K, has a crucial role in this process, as does a family of proteins known as Rab GTPases. VPS34 is conserved in all eukaryotes, regulating core “housekeeping” signalling pathways, such as autophagy and endosomal trafficking. The different Rab proteins recruit VPS34 to the correct location within the cell so that it can carry out its different roles.

VPS34 functions within two related protein complexes, VPS34 complex I and VPS34 complex II. These complexes share three common subunits – VPS34 itself, VPS15 and BECLIN1 – but differ in their fourth subunit. VPS34 complex I, which is crucial for autophagy, also contains a protein called ATG14L, whereas VPS34 complex II – which is involved in endosomal trafficking – has a protein called UVRAG as its fourth subunit (Figure 1).

**Figure 1.**
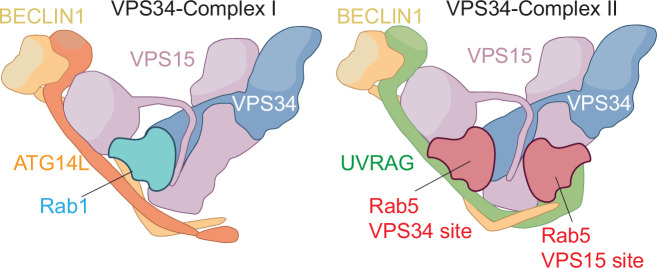
Differences between VPS34 complex I and VPS34 complex II. VPS34 complex I (left) contains four subunits: VPS34 (blue), VPS15 (mauve), BECLIN1 (gold) and ATG14L (orange). VPS34 complex II (right) also contains VPS34, VPS15 and BECLIN1, along with UVRAG as the fourth subunit. Rab1 binds to the VPS34 subunit in complex I, while Rab5 binds to a very similar site in the VPS34 subunit in complex II. Špokaitė et al. show how the fourth subunit in each complex – ATG14L for complex I, UVRAG for complex II – reshapes the Rab-binding pocket in the VPS34 subunit to confer specificity for Rab1 or Rab5. Moreover, Špokaitė et al. discovered a second binding site for Rab5 in the VPS15 subunit in complex II.

This raises a central question: if these two complexes are so similar, how can they uniquely engage with different Rab proteins? Now, in eLife, Roger Williams and colleagues at the MRC Laboratory of Molecular Biology – including Saulė Špokaitė as first author, Yohei Ohashi, Maxime Bourguet and Antonie Dessus – report the results of experiments that help to answer this question (Špokaitė et al., 2026).

Previous work showed that Rab1 binds and activates VPS34 complex I, whereas Rab5 binds and activates VPS34 complex II at an almost identical site (Cook et al., 2025; Tremel et al., 2021). Špokaitė et al. started by following up on a puzzling observation: a mutation in VPS34 designed to disrupt interactions between the enzyme and the Rab proteins had completely different effects in Rab1 and Rab 5. This mutation prevented VPS34 complex I binding to Rab1 but, surprisingly, it strengthened the interaction between VPS34 complex II and Rab5. The researchers carried out a series of experiments to locate the Rab5 binding site in VPS34 complex II.

When Špokaitė et al. used cryo-electron microscopy to determine the structure of Rab5 bound to the VPS34 mutant, they unexpectedly found a second Rab5 bound to the VPS15 subunit (Figure 1). Further experiments on wild-type VPS34 complex II and a different mutant – using both cryo-electron microscopy and a technique called hydrogen-deuterium exchange mass spectrometry – confirmed that VPS34 complex II can engage with two Rab5 molecules at distinct binding sites. Moreover, biochemical assays showed that both sites are required for full activation of VPS34 complex II by Rab5.

The researchers then returned to the question of why the VPS34 enzyme binds Rab1 in complex I, but Rab5 in complex II, despite the two binding sites being so similar. The answer lies in the fourth subunit in each complex. ATG14L leaves the binding site more open in complex I, favouring Rab1 binding, whereas UVRAG closes the site, which promotes Rab5 binding. Although neither Rab1 or Rab5 make contact with ATG14L or UVRAG, in each case the fourth subunit works to reshape the Rab binding site in VPS34. In this way conformational dynamics, rather than the Rab proteins themselves, ensures that Rab1 only binds to complex I and Rab5 only binds to complex II.

Surprisingly, the new binding site for Rab5 in VPS15 is highly conserved, whereas the binding site in VPS34 is absent in yeast. Moreover, mutations in the VPS15 binding site in yeast lead to defects in endocytic trafficking, indicating that the newly discovered Rab5 binding site is essential for proper functioning of VPS34 complex II in endosomal sorting across many different eukaryotes.

The increase in the number of Rab5 binding sites in VPS34 complex II in higher eukaryotes suggests that it may be possible to sense Rab5 surface density, allowing even tighter control of the class III PI3K signalling pathway that links VPS34 and Rab signalling. Although questions remain about the role of Rab5 engagement of VPS34 complex II in human cells, the work of Špokaitė et al. provides an essential mechanistic foundation for exploring how Rab proteins help to ensure that chemical reactions take place where they should in cells.
